# Mucosal microbiome is predictive of pediatric Crohn’s disease across geographic regions in North America

**DOI:** 10.12688/f1000research.108810.1

**Published:** 2022-02-08

**Authors:** Rajesh Shah, Kristi Hoffman, Lee Denson, Subramaniam Kugathasan, Richard Kellermayer

**Affiliations:** 1 Suite 200, Baylor Health Care System, Austin, Texas, 78735, USA; 2 Baylor College of Medicine, Houston, USA; 3 Cincinnati Children's Hospital Medical Center, Cincinnati, USA; 4 Emory University, Atlanta, USA

**Keywords:** Crohn’s disease, microbiome, inflammatory bowel disease, machine learning

## Abstract

**Background:** Patients with Crohn’s disease (CD) have an altered intestinal microbiome, which may facilitate novel diagnostic testing. However, accuracy of microbiome classification models across geographic regions may be limited. Therefore, we sought to examine geographic variation in the microbiome of patients with CD from North America and test the performance of a machine learning classification model across geographic regions.

**Methods:** The RISK cohort included 447 pediatric patients with CD and 221 non-inflammatory bowel disease controls from across North America. Terminal ileum, rectal and fecal samples were obtained prior to treatment for microbiome analysis. We divided study sites into 3 geographic regions to examine regional microbiome differences. We trained and tested the performance of a machine learning classification model across these regions.

**Results:** No differences were seen in the mucosal microbiome of patients with CD across regions or in either the fecal or mucosal microbiomes of controls. Machine learning classification algorithms for patients with CD performed well across regions (area under the receiver operating characteristic curve [AUROC] range of 0.85-0.91) with the best results from terminal ileum.

**Conclusions:** This study demonstrated the feasibility of microbiome based diagnostic testing in pediatric patients with CD within North America, independently from regional influences.

## Introduction

Currently, our understanding is the intestinal microbiome plays a role in the pathogenesis of inflammatory bowel disease (IBD), ^1^ and specifically Crohn’s disease (CD). ^2^ The RISK consortium found significant differences in the taxonomy of the mucosal and fecal microbiomes of pediatric, treatment naïve patients with CD compared to non-IBD controls. ^3^ Similar results were demonstrated in a longitudinal study of adult patients with IBD with an emphasis on disruption of the microbiome during periods of disease activity. ^4^

Based on an altered microbiome composition in patients with CD, microbiome signatures may be utilized as a diagnostic biomarker. From the original RISK publication, ^3^ the addition of microbiome data to clinical information improved the performance of their classification models for CD. Similarly, Pascal *et al*. showed microbiome classification models for CD were accurate and performed well across 4 countries in Europe (Spain, Belgium, the UK and Germany). ^2^

Recent data suggested that geographic bias, however, may limit the validity of microbiome based diagnostic models. He *et al*. studied 7,009 individuals from 1 Chinese province with 14 districts to determine regional differences in the microbiome. ^5^ They found strong associations between microbiome composition and host district, which translated into decreased model performance when classifying metabolic diseases across districts. However, they acknowledged that other diseases, such as CD, could not be studied due to a limited sample size. Therefore, we sought to examine differences in the intestinal microbiome of pediatric patients with CD by region and to determine if geographic bias hinders the performance of a machine learning classification model across regions in North America.

## Methods

### Participants

A *post hoc* analysis of the RISK cohort was performed. The RISK cohort was a multicenter study that enrolled treatment naïve pediatric patients aged 3 to 17 years with CD and non-IBD controls from 28 sites with the United States and Canada from 2008 to 2012. ^3^ All patients had symptoms suggestive of CD, including abdominal pain or diarrhea that prompted evaluation with a colonoscopy with biopsies from the terminal ileum and rectum. A subset of patients also provided fecal samples. Patients were either diagnosed with CD, based on endoscopic appearance and histology, or a non-inflammatory etiology for their symptoms, which served as the non-IBD controls. Full inclusion and exclusion criteria for the RISK cohort have been described in the original publication. ^3^ In total, 447 patients with CD and 221 non-IBD controls were included in the original publication and they provided a total of 1,321 samples, including 630 ileal, 387 rectal and 304 fecal samples.

### Ethical considerations

IRB approval was not required for this study, as deidentified data was used and consent was previously obtained from participants when they enrolled in the RISK cohort study.

### Statistical analysis

**Figure 1.**
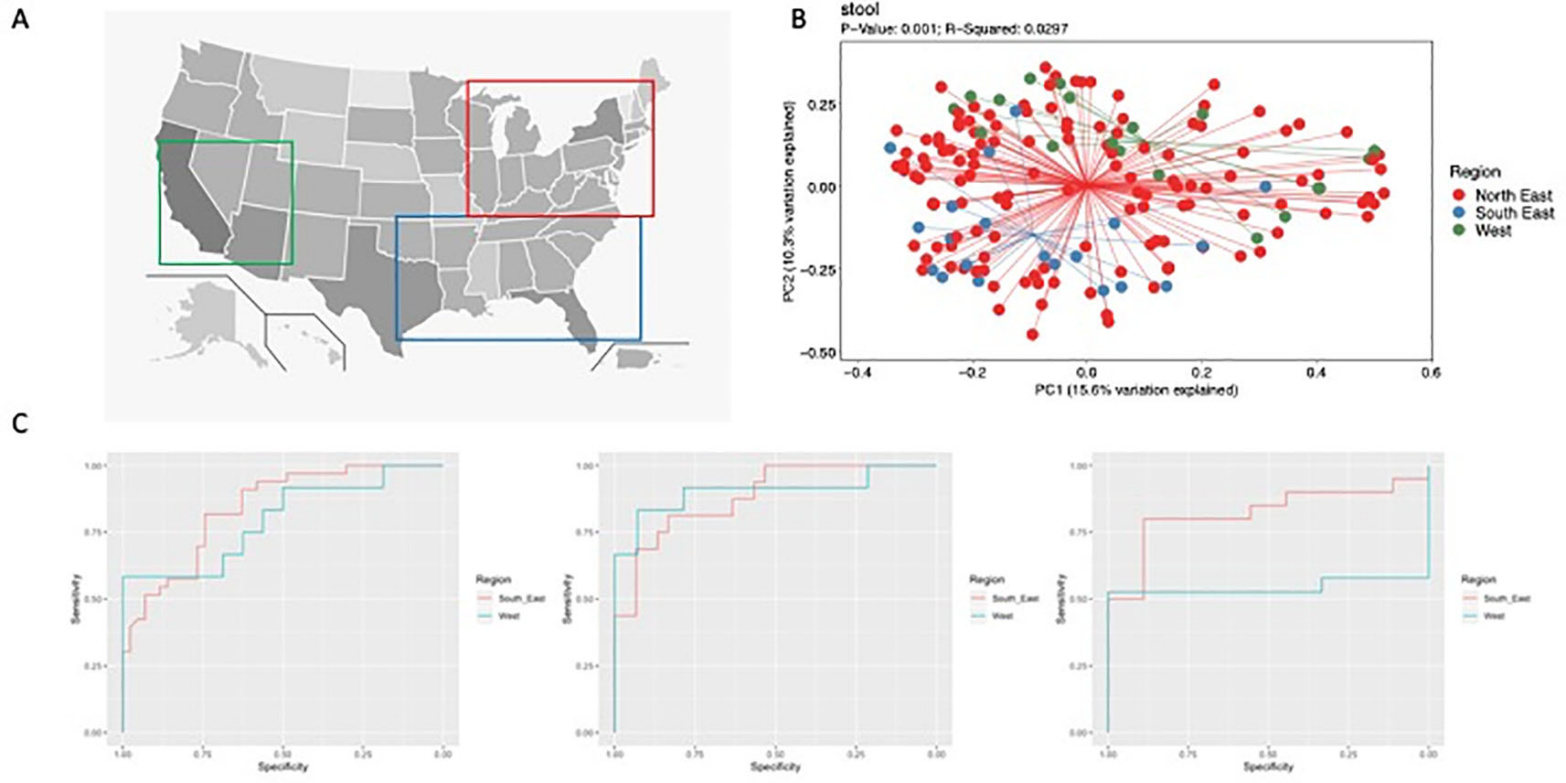
A) Map of the United States with squares indicating the arbitrarily defined North East (Red), South East (Blue) and West (Green) regions, B) Principal Component Analysis of weighted Bray Curtis metric for feces from patients with CD by region (PERMANOVA), C) Receiver operating characteristic (ROC) curves for a CD prediction model trained using North East data and tested on South East and West data for ileum, rectum and feces, from left to right.

Age at diagnosis, sex, race, disease phenotype, and treatment center were examined. To evaluate the influence of geography on microbiome composition, we grouped the treatment centers into 3 subjective regions based on overall geography (North-East, South-East and West, Figure 1A).

*16Sv4* rRNA gene analysis was performed in the original cohort study using the Illumina MiSeq platform. For our analysis, the original biom table was obtained and rarefied to 3,441 sequences per sample. This rarefaction depth was chosen to retain the maximum number of samples and preserve the most amount of sequencing data per sample. The alpha and beta diversity and taxonomic composition of the terminal ileum, rectum, and fecal microbiomes were evaluated using the ATIMA interface version 1.0 available through the Baylor College of Medicine Alkek Center for Metagenomics and Microbiome Research. ATIMA is a graphic user interface that allows users to provide a biom table and mapping file for microbiome analysis. To adjust for potential confounding, MaasLin was used to control for variations in age at diagnosis, sex, race, sample type and geographic region. ^6^

Finally, we sought to develop a machine learning model to evaluate the accuracy of a microbiome model to identify patients with CD across different regions. A random forest machine learning model was trained on patients from the North-East and tested in the South-East and West using the R package healthcare.ai version 2.5.0 with the default settings. The healthcare.ai package is an open-source R package that allows for data cleaning, manipulation, imputation, tuning of models and evaluation of model performance. Visualization of model performance with AUROC metrics was done using the R package pROC version 1.18.0.

## Results

Based on the terminal ileum biopsies retained after rarefaction, we included 227 patients with CD and 165 controls with a mean age of 12.2 and 12.1 years, respectively. Approximately half of patients with CD and controls were male (58.6% and 53%, respectively) and a larger proportion of patients with CD were Caucasian compared to controls (78.9% and 68.7%, respectively). Since microbiome composition can be influenced by the presence of stricturing/fistulizing disease ^7^ and these patients present less of a diagnostic challenge, they were excluded from our analysis to create a consistent population with an inflammatory phenotype. After separating into regions, 182 patients were in the North-East, 33 in the South-East and 12 in the West with CD, and 106 patients in the North-East, 43 in the South-East and 16 in the West without IBD.

For patients with CD, no significant differences were found in alpha and beta diversity of the ileal and rectal mucosal microbiome by geography. However, PCoA plots of unweighted and weighted beta diversity ( Figure 1B) determined through the Bray Curtis metric revealed significant differences in fecal samples. In controls, no significant differences were found in alpha and beta diversity of the ileum, rectum or fecal samples. In the South-East, patients with CD had a relative increase in Fusobacteria and Bacteroidetes with a decrease in Actinobacteria and Firmicutes in fecal samples compared to the other 2 regions. This corresponded to an increase in the genera *Bacteroides* and *Fusobacterium* with a decrease in *Bifidobacterium* and *Lactobacillus.* However, after adjustment with MaasLin, *Erwinia* was the only genus associated with geographical variation in patients with CD. Specifically, fecal samples from CD patients in the South-East had increased abundance of *Erwinia* compared to other geographic regions in North America (q=0.04).

Random forest models across sample types performed well ( Figure 1C). The best performance occurred with ileal samples (North-East AUROC 0.89, South-East AUROC 0.85 and West AUROC 0.91). The rectal (North-East AUROC 0.87, South-East AUROC 0.83, West AUROC 0.76) and fecal (North-East AUROC 0.82, South-East AUROC 0.85, West AUROC 0.74) samples performed well, but experienced decreased performance in the West. Comparing the models, those for ileum and rectum shared OTUs discriminating CD, which included members of the Lachnospiraceae and Clostridiaceae families and the genus *Blautia.* Intriguingly, ileal biopsies and fecal samples shared top CD-discriminating OTUs from the Erysipelotrichaceae family and *Haemophilus* genus, which were not present between rectal biopsies and fecal samples.

## Discussion

Our results indicate that CD influences mucosal microbiome composition to a greater extent than geography in pediatric patients from North America. Machine learning classification models performed well across the regions, despite minor differences in the fecal microbiome of CD patients. Differences in microbiome composition are known to vary across populations in healthy cohorts ^8^^,^^9^ and in patients with metabolic syndrome. ^5^ Yatsunenko *et al*. showed Westernization may influence fecal microbiome composition by comparing samples from subjects in the US, Venezuela and Malawi. ^8^ Similar patterns were seen by Pasolli *et al*. when they examined metagenomes from 9,428 samples from 32 countries and noted significant differences in the metagenomes of Western populations. ^9^ Together, these studies demonstrated microbiome composition varies across populations, however, they did not address microbiome differences within countries. To that end, He *et al*. studied a single province in China and noted differences in microbiome composition between its districts. ^5^ This suggested, as has been previously reviewed, that a vast number of environmental factors may play a role in shaping the microbiome and may limit the accuracy of microbiome classification models. ^10^

Overall, our classification models performed well across regions and is consistent with prior reports. Using 2,045 fecal samples taken from patients with IBD and non-IBD controls across 4 European countries, Pascal *et al*. showed that a microbial signature could be used to discriminate patients with CD from non-IBD controls with an overall sensitivity of 80% and specificity of 94%. ^2^ In a separate cohort, Franzosa *et al*. used metagenomics and metabolomics to distinguish IBD patients from non-IBD patients also with high accuracy. ^11^ Our findings in pediatric CD are consistent with these results and demonstrate the feasibility of using microbiome classification models to accurately diagnose CD without geographic bias within North America.

Despite the limitations of our study, our classification models performed well. We were unable to adjust for additional confounders of microbiome composition, such as diet and supplement intake. ^12^ However, even without this information, our models based on ileal biopsies performed well. Additionally, we noted a decrease in model performance for fecal samples and in the West, but this may be linked to a smaller sample size, which is known to hinder the performance of machine learning models. Further work with larger cohorts and different control groups will be needed to fully determine whether microbiome machine learning models can support the diagnosis of CD in children without geographical bias, and if non-invasive testing with fecal samples is feasible.

In summary, machine learning models can distinguish patients with CD from non-IBD controls without geographic bias in North America. Further development of microbiome machine learning models to diagnose CD may be warranted.

## Data availability

### Underlying data

NCBI BioProject: human gut metagenome. Accession number PRJNA237362; https://identifiers.org/NCBI/bioproject:PRJNA237362.

The underlying clinical data used for this study is available through the RISK consortium. Consortium approval was required to access de-identified patient data and requests can be placed through the Crohn’s and Colitis Foundation IBD Plexus Initiative ( www.crohnscolitisfoundation.org/research/granst-fellowships/ibd-plexus).

## References

[1] Huttenhower C Kostic AD Xavier RJ : Inflammatory Bowel Disease as a Model for Translating the Microbiome. *Immunity.* 2014;40(6):843–854. 10.1016/j.immuni.2014.05.013 24950204 PMC4135443

[2] Pascal V Pozuelo M Borruel N : A microbial signature for Crohn’s disease. *Gut.* 2017;66(5):813–822. 10.1136/gutjnl-2016-313235 28179361 PMC5531220

[3] Gevers D Kugathasan S Denson LA : The Treatment-Naive Microbiome in New-Onset Crohn’s Disease. *Cell Host Microbe.* 2014;15(3):382–392. 10.1016/j.chom.2014.02.005 24629344 PMC4059512

[4] Lloyd-Price J Arze C Ananthakrishnan AN : Multi-omics of the gut microbial ecosystem in inflammatory bowel diseases. *Nature.* 2019;569(7758):655–662. 10.1038/s41586-019-1237-9 31142855 PMC6650278

[5] He Y Wu W Zheng H-M : Regional variation limits applications of healthy gut microbiome reference ranges and disease models. *Nat. Med.* 2018;24(10):1532–1535. 10.1038/s41591-018-0164-x 30150716

[6] Morgan XC Tickle TL Sokol H : Dysfunction of the intestinal microbiome in inflammatory bowel disease and treatment. *Genome Biol.* 2012;13(9):R79. 10.1186/gb-2012-13-9-r79 23013615 PMC3506950

[7] Kugathasan S Denson LA Walters TD : Prediction of complicated disease course for children newly diagnosed with Crohn’s disease: a multicentre inception cohort study. *Lancet.* 2017;389:1710–1718. 10.1016/s0140-6736(17)30317-3 28259484 PMC5719489

[8] Yatsunenko T Rey FE Manary MJ : Human gut microbiome viewed across age and geography. *Nature.* 2012;486(7402):222–227. 10.1038/nature11053 22699611 PMC3376388

[9] Pasolli E Asnicar F Manara S : Extensive Unexplored Human Microbiome Diversity Revealed by Over 150,000 Genomes from Metagenomes Spanning Age, Geography, and Lifestyle. *Cell.* 2019;176:649–662.e20. 10.1016/j.cell.2019.01.001 30661755 PMC6349461

[10] Gupta VK Paul S Dutta C : Geography, Ethnicity or Subsistence-Specific Variations in Human Microbiome Composition and Diversity. *Front. Microbiol.* 2017;8:1162. 10.3389/fmicb.2017.01162 28690602 PMC5481955

[11] Franzosa EA Sirota-Madi A Avila-Pacheco J : Gut microbiome structure and metabolic activity in inflammatory bowel disease. *Nat. Microbiol.* 2019;4(2):293–305. 10.1038/s41564-018-0306-4 30531976 PMC6342642

[12] Lewis JD Chen EZ Baldassano RN : Inflammation, Antibiotics, and Diet as Environmental Stressors of the Gut Microbiome in Pediatric Crohn’s Disease. *Cell Host Microbe.* 2015;18(4):489–500. 10.1016/j.chom.2015.09.008 26468751 PMC4633303

