# Supplementary material for: Mucosal microbiome is predictive of pediatric Crohn’s disease across geographic regions in North America
Source: F1000Res. 2023 Jan 4;11:156. Originally published 2022 Feb 8. [Version 2] doi: 10.12688/f1000research.108810.2 (PMC9860194; doi:10.12688/f1000research.108810.2)
Supplement: Peer review history

# Peer review: Reviewer response for version 2

**DOI:** 10.5256/f1000research.142172.r158956

**Published:** 2023-01-20

**Authors:** Ranko Gacesa

**Affiliations:** 1 Department of Gastroenterology and Hepatology, University Medical Centre Groningen, University of Groningen, Groningen, The Netherlands

Authors have included supplementary material with codes used to train machine learning classifiers. I have no further comments.

Is the work clearly and accurately presented and does it cite the current literature?

Yes

If applicable, is the statistical analysis and its interpretation appropriate?

Yes

Are all the source data underlying the results available to ensure full reproducibility?

Partly

Is the study design appropriate and is the work technically sound?

Yes

Are the conclusions drawn adequately supported by the results?

Yes

Are sufficient details of methods and analysis provided to allow replication by others?

Partly

Reviewer Expertise:

Bioinformatics, Microbiome, Artificial intelligence, Inflammatory Bowel Disease

I confirm that I have read this submission and believe that I have an appropriate level of expertise to confirm that it is of an acceptable scientific standard.

# Peer review: Reviewer response for version 2

**DOI:** 10.5256/f1000research.142172.r158957

**Published:** 2023-01-05

**Authors:** Jonathan Braun

**Affiliations:** 1 F. Widjaja IBD Institute, Cedars Sinai Medical Center, Los Angeles, CA, 90095-1732, USA

The revision (RF model) is helpful. Good addition.

Is the work clearly and accurately presented and does it cite the current literature?

Yes

If applicable, is the statistical analysis and its interpretation appropriate?

Yes

Are all the source data underlying the results available to ensure full reproducibility?

Yes

Is the study design appropriate and is the work technically sound?

Yes

Are the conclusions drawn adequately supported by the results?

Yes

Are sufficient details of methods and analysis provided to allow replication by others?

No

Reviewer Expertise:

Immunology and microbiome in IBD

I confirm that I have read this submission and believe that I have an appropriate level of expertise to confirm that it is of an acceptable scientific standard.

# Peer review: Reviewer response for version 1

**DOI:** 10.5256/f1000research.120239.r150775

**Published:** 2022-10-14

**Authors:** Ranko Gacesa

**Affiliations:** 1 Department of Gastroenterology and Hepatology, University Medical Centre Groningen, University of Groningen, Groningen, The Netherlands

Authors examined consistency of microbiome-trained machine learning models for prediction of Crohn's disease across US geography. They demonstrated that models trained on one of geographic regions perform well on ileal samples, but perform less consistently for fecal and rectal samples.

 Study is relevant (as the loss of predictive power of microbiome models due to geographic variation was demonstrated before in the Chinese population) and methodologically sound. The study would, however, benefit from sharing of used codes, produced models and other technical details to facilitate the reproducibility and comparison with other, related, studies.

Is the work clearly and accurately presented and does it cite the current literature?

Yes

If applicable, is the statistical analysis and its interpretation appropriate?

Yes

Are all the source data underlying the results available to ensure full reproducibility?

Partly

Is the study design appropriate and is the work technically sound?

Yes

Are the conclusions drawn adequately supported by the results?

Yes

Are sufficient details of methods and analysis provided to allow replication by others?

Partly

Reviewer Expertise:

Bioinformatics, Microbiome, Artificial intelligence, Inflammatory Bowel Disease

I confirm that I have read this submission and believe that I have an appropriate level of expertise to confirm that it is of an acceptable scientific standard, however I have significant reservations, as outlined above.

# Response

**Published:** 2022-11-08

**Authors:** Rajesh Shah

**Affiliations:** Baylor Health Care System, USA

We are happy to provide the underlying code that was used to generate the data for this study in a supplement to facilitate reproducibility.

# Peer review: Reviewer response for version 1

**DOI:** 10.5256/f1000research.120239.r150774

**Published:** 2022-10-05

**Authors:** Jonathan Braun

**Affiliations:** 1 F. Widjaja IBD Institute, Cedars Sinai Medical Center, Los Angeles, CA, 90095-1732, USA

Regional contribution to microbiome composition is an important and less-assessed factor in refining conclusions about disease association and microbiome. This study is useful in addressing this question in the context of US regions and the landmark RISK cohort study of Crohn's disease.

 My issues with the paper are minor. It references several assessments with negative associations, for which data is not shown. It is fine to do so, but the methods should be briefly noted: what was the alpha diversity metric(s); and, is the unweighted beta diversity (noted in context of Figure 1, which is weighted) a data-not-shown finding, or a clean-up needed in the text?

 Figure 1B has "lines" connecting dots in the PCoA plot. What do these lines represent?

Is the work clearly and accurately presented and does it cite the current literature?

Yes

If applicable, is the statistical analysis and its interpretation appropriate?

Yes

Are all the source data underlying the results available to ensure full reproducibility?

Yes

Is the study design appropriate and is the work technically sound?

Yes

Are the conclusions drawn adequately supported by the results?

Yes

Are sufficient details of methods and analysis provided to allow replication by others?

No

Reviewer Expertise:

Immunology and microbiome in IBD

I confirm that I have read this submission and believe that I have an appropriate level of expertise to confirm that it is of an acceptable scientific standard.

# Response

**Published:** 2022-10-11

**Authors:** Rajesh Shah

**Affiliations:** Baylor Health Care System, USA

We greatly appreciate the comments and favorable review. To address the raised points, we limited the presentation of negative findings to allow us to focus on positive findings. In terms of the metrics used, we used the Shannon metric to measure alpha diversity and the Bray-Curtis metric for beta diversity. The lines presented in Figure 1B refer to the centroid of each Region.
